# Platelet-to-Lymphocyte Ratio Is Associated With Favorable Response to Neoadjuvant Chemotherapy in Triple Negative Breast Cancer: A Study on 120 Patients

**DOI:** 10.3389/fonc.2021.678315

**Published:** 2021-07-20

**Authors:** Sejdi Lusho, Xavier Durando, Marie-Ange Mouret-Reynier, Myriam Kossai, Nathalie Lacrampe, Ioana Molnar, Frederique Penault-Llorca, Nina Radosevic-Robin, Catherine Abrial

**Affiliations:** ^1^ Clermont Auvergne University, INSERM U1240 “Molecular Imaging and Theranostic Strategies”, Centre Jean Perrin, Clermont-Ferrand, France; ^2^ Delegation for Clinical Research and Innovation, Centre Jean Perrin, Clermont-Ferrand, France; ^3^ Centre for Clinical Investigation, INSERM U501, Clermont-Ferrand, France; ^4^ Department of Medical Oncology, Centre Jean Perrin, Clermont-Ferrand, France; ^5^ Department of Pathology, Centre Jean Perrin, Clermont-Ferrand, France

**Keywords:** neoadjuvant chemotherapy, pathologic complete response (pCR), peripheral blood counts, platelet-to-lymphocyte ratio (PLR), triple negative breast cancer (TNBC), tumor-infiltrating lymphocytes (TILs), distant recurrence

## Abstract

**Introduction:**

Triple negative breast cancer (TNBC) is highly heterogeneous, but still most of the patients are treated by the anthracycline/taxane-based neoadjuvant therapy (NACT). Tumor-infiltrating lymphocytes (TILs) are a strong predictive and prognostic biomarker in TNBC, however are not always available. Peripheral blood counts, which reflect the systemic inflammatory/immune status, are easier to obtain than TILs. We investigated whether baseline white cell or platelet counts, as well as, Neutrophil-to-Lymphocyte Ratio (NLR) or Platelet-to-Lymphocyte Ratio (PLR) could replace baseline TILs as predictive or prognostic biomarkers in a series of TNBC treated by standard NACT.

**Patients and Methods:**

One hundred twenty patients uniformly treated by FEC/taxane NACT in a tertiary cancer care center were retrospectively analyzed. The presence of pathological complete response (pCR: ypT0/Tis, ypN0) or the presence of pCR and/small residual disease (ypT0/Tis/T1ab, ypN0) were considered as good responses in data analysis. Baseline/pre-NACT blood count, NLR, PLR and TILs were evaluated as predictors of response, distant recurrence rate and distant recurrence-free survival (DRFS).

**Results:**

TILs ≥30% and ≥1.5% were best predictors of pCR and distant recurrence risk, respectively (p = 0.007, p = 0.012). However, in this cohort, pCR status was not significantly associated with recurrence. Only the ensemble of patients with pCR and small residual disease had lower recurrence risk and longer survival DRFS (p = 0.042, p = 0.024, respectively) than the rest of the cohort (larger residual disease). The only parameter which could predict the pCR/small residual disease status was PLR: patients with values lower than 133.25 had significantly higher chance of reaching that status after NACT (p = 0.045). However, no direct correlation could be established between baseline PLR and metastatic recurrence. No correlation either was found between TIL and individual blood counts, or between TILs and NLR or PLR.

**Conclusion:**

In this cohort, TILs retained their pCR predictive value; however PLR was a better predictor of the ensemble of responses which had good outcome in terms of less distant recurrences or longer DRFS (pCR or small residual disease). Thus, baseline PLR is worth further, prospective investigation together with baseline TILs, as it might indicate a good TNBC response to NACT when TILs are unavailable.

## Introduction

Treatment of triple negative breast cancer (TNBC) is currently one of the biggest challenges in oncology. TNBC is characterized by absence or very low expression of estrogen and progesterone receptor in tumor cells, associated with absence of *ERBB2* gene amplification. TNBC accounts for 10–15% of all breast cancers and tends to be more common among younger women and women carrying a *BRCA1* gene mutation (1, 2). As a group, this breast cancer type has a poorer prognosis compared to other breast cancer molecular subtypes; however triple negative tumors are very heterogeneous in terms of clinical behavior. In spite of this heterogeneity, therapeutic approach to TNBC is still limited to neoadjuvant chemotherapy (NACT), post-NACT surgery and adjuvant radiation therapy as no targeted therapies are available for the early phase of the disease (3). Distant recurrences are frequent within the first 5 years after the initial treatment completion (2) and the median survival of patients with metastatic disease is shorter than 15 months (1, 4, 5). Therefore, it is necessary to identify, as early as possible during the disease course, the biomarkers useful to determine the risk of metastatic relapse for a given patient. This can be used to monitor the disease evolution under NACT and to adapt the therapeutic strategy for high-risk patients.

In TNBC, a pathologic complete response (pCR) to NACT is considered to be the most powerful predictor of good disease-free survival (DFS) and overall survival (OS) (6, 7), so it is used as the principal surrogate endpoint for patient outcome. However, new data suggest that TNBC patients with small residual tumors after NACT may have similar outcome as patients who reach pCR. Symmans et al. showed excellent prognoses for both TNBC patients with pCR and those having score 1 of the Residual Cancer Burden (RCB), after anthracycline/taxane-based NACT (8, 9). Similar results were obtained by Sharma et al. in TNBC patients treated by neoadjuvant carboplatin-docetaxel (10). Other studies have reported that pCR and near-pCR to NACT were significantly associated with better DFS and OS, and that the presence of a small residual tumor after NACT does not adversely affect TNBC outcome (11). Therefore, the presence of small residual post-NACT disease, together with pCR, has been increasingly considered as a favorable response to NACT and as an endpoint in clinical and translational TNBC trials. In line with this, biomarkers which could predict such a response of a given TNBC to NACT, are actively sought for.

In the recent years, parameters reflecting the immune response to cancer, either within the tumor site, or in the circulation (blood), have been shown to have predictive or/and prognostic value in oncology. For example, high baseline numbers of tumor-infiltrating lymphocytes (TILs) are associated with higher rates of TNBC pCR to NACT (12, 13). Other studies have demonstrated a correlation between high levels of TILs in post-NACT TNBC residual tumors and improved patient survival (14, 15). However, a TIL number is not routinely available to many breast oncologists, for several major reasons: need for a highly skilled pathologist to determine it, breast cancer diagnostic biopsy performed outside the institution which will treat the patient or small biopsy size not representative for TIL assessment although sufficient for breast cancer diagnosis. Moreover, there is a degree of inherent error in TIL number evaluation, especially when TIL quantity is moderate. For all this, either other biomarkers of the patient or/and tumor immune status are needed, easier to assess and more widely implemented in the clinic than TILs, or the efforts to meet the conditions for correct TIL assessment in routine breast oncology practice should be significantly intensified.

Numerous studies have suggested that parameters derived from peripheral blood counts, such as neutrophil-to-lymphocyte ratio (NLR) and platelet-to-lymphocyte ratio (PLR), could predict response to treatment and prognosis in several cancers, including breast cancer. Peripheral white blood counts are indicators of systemic inflammation, which have been shown to have an impact on cancer development and metastatic progression (16). Indeed, high values of NLR and/or PLR have been shown to correlate with poor prognosis in various types of cancer, such as gastric, colorectal, ovarian, pancreatic and lung cancer (17–22).

Several studies have reported that low PLR is associated to higher pCR rates, and better DFS/OS in breast cancer patients treated with NACT (23, 24). Other studies have reported similar results for both PLR and NLR, either separately (16, 25) or combined (6). Interestingly, a relatively small number of such studies have been conducted on TNBC; most of them have shown stronger prognostic value for NLR than for PLR (26, 27).

In this study, we investigated whether the pre-treatment neutrophil, lymphocyte, monocyte or platelet count, as well as NLR or PLR provides a better information than TILs about TNBC response to NACT or/and distant recurrence-free survival (DRFS), in a retrospective monocentric cohort uniformly treated by a standard-of-care anthracycline/taxane regimen.

## Patients and Methods

### Ethics Statement

Patients were informed of the investigational nature of this study and were given the possibility to oppose to their data being used. All the data analyzed in the scope of this paper came from patients that were not opposed to such use.

The ethics approval of the study was obtained from the Ethical Committee of the Clinical Investigation Centre of the Rhone-Alpes-Auvergne region, Grenoble, France; approval number: IRB 5921.

### Patients

Patient data were retrieved from the TNBC database of Centre Jean Perrin. Medical records of the patients carrying an early TNBC treated by NACT from 2008 to 2019 were reviewed and the patients were selected for this study according to three main criteria: (1) histologically proven non-metastatic TNBC at diagnosis, (2) anthracycline/taxane-based NACT only (no other drugs, no neoadjuvant radiotherapy) and (3) available blood counts before NACT, for evaluation of NLR and PLR.

### Data Collection

Clinical, histological and biological data of the selected patients have been previously collected and entered, in a pseudonymized fashion, into the TNBC database of Centre Jean Perrin, and were extracted for the present analysis.

### Blood Counts

Blood tests were performed on the day before the start of the NACT, in the reference university laboratory serving Centre Jean Perrin. NLR was defined as the ratio between the absolute number of neutrophils (ANC, number of cells × 10^12^/L) and the absolute number of lymphocytes (ALC, number of cells × 10^12^/L). PLR was defined as the ratio between the absolute number of platelets (APC, number of platelets × 10^12^/L) and ALC.

### Pathological Assessments

The triple negative status of all biopsies and surgical specimens was determined by immunohistochemistry (IHC). Hormone receptor status was considered negative if 0–9% of tumor cells had nuclear receptor staining (28, 29), in conformity with the institution practices at the Centre Jean Perrin and in France in general. HER2 negativity was defined as score 0 or 1+ on IHC or the absence of *ERBB2* gene amplification by *in situ* hybridization, if the HER2 IHC score was 2 (30).

pCR was defined as the absence of residual invasive tumor in the breast and the lymph nodes (ypT0/T*is* ypN0) and the absence of metastases (M0) (31).

Due to a lack of tissue blocks, we were not able to determine the RCB score. Therefore, we defined small residual disease, for purpose of this research, as all ypT1a ypN0 and ypT1b ypN0 responses to NACT. Further, we categorized the patients in two groups. First, small or absent residual disease group (hereafter defined as Group 1) included patients with pCR and small residual disease. The second group (hereafter defined as Group 2) included all other patients: small residual disease with lymph node involvement and large residual disease (ypT1c or greater) regardless of the lymph node status.

The amount of TILs in pre-NACT tumor biopsies was assessed according to recommendations of the International Immuno-Oncology Biomarker Working Group on Breast Cancer (32). In statistical analysis, TILs were taken into account as a continuous variable and as a binary variable by several prespecified cut-offs (5, 10, 30 and 50%), similarly to the cut-offs reported in the literature (33).

### Study Endpoints

The primary goal of this study was to determine whether baseline NLR and/or PLR better predict TNBC response (pCR or [pCR + small residual disease]) to NACT than baseline TILs. The secondary goal was to determine whether baseline NLR and/or PLR have a greater prognostic value than TILs, for patient distant recurrence risk or DRFS.

### Statistical Analysis

Statistical analysis was performed using the R software, version 3.6.1 (R-Project, GNU GPL, http://cran.r-project.org/). The relationship between categorical variables was evaluated by contingency matrices and Fisher’s exact test, whereas the Wilcoxon–Mann–Whitney test, Student’s test and ROC curves were used to evaluate the relationship between continuous and categorical variables. The relationship between continuous variables and DRFS was evaluated using ROC curves and Cox regression models, Kaplan–Meier survival curves, and log-rank test.

## Results

### Patient Characteristics

**Figure 1:**
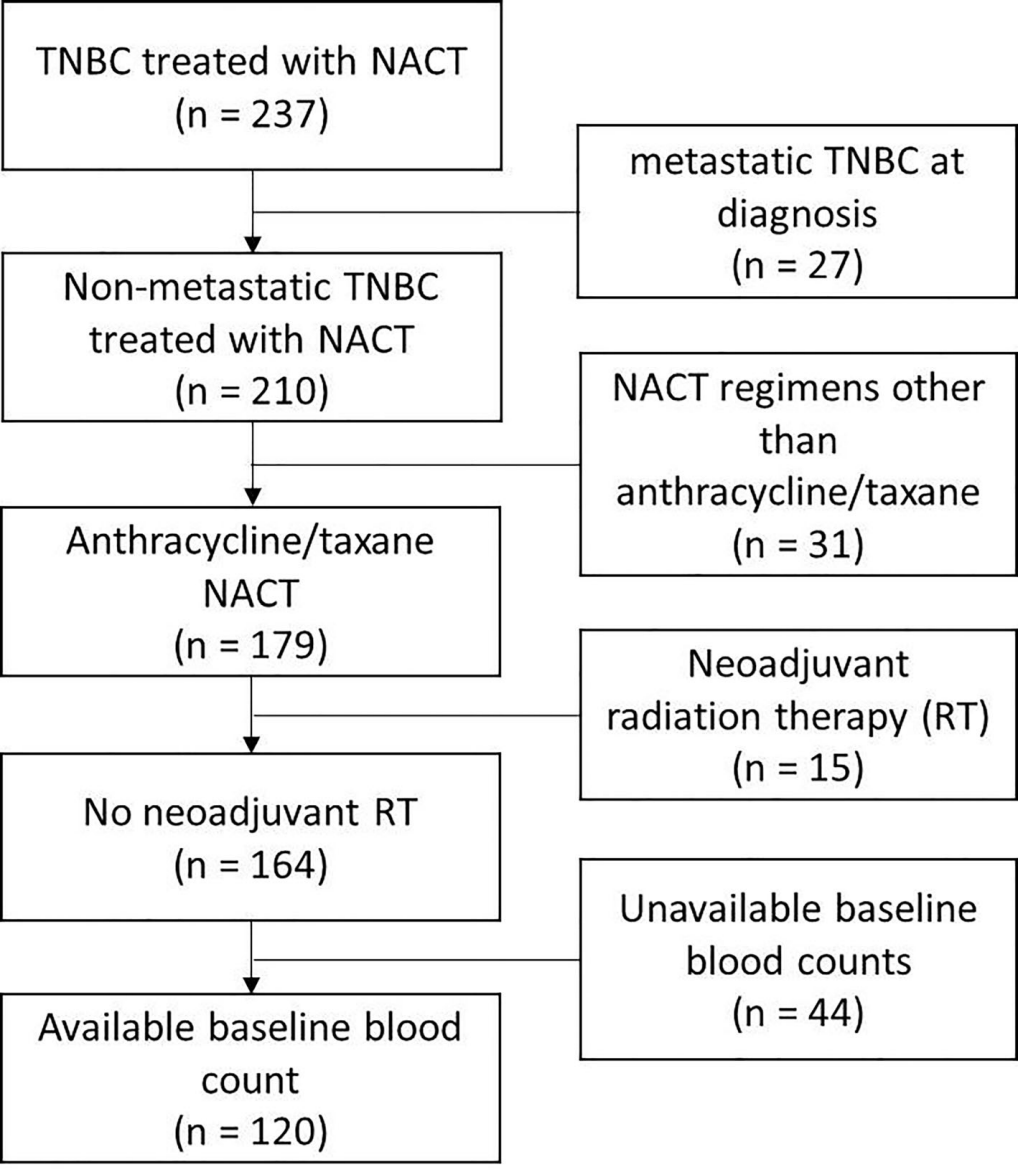
Patient selection flowchart.

We identified 237 TNBC patients treated with NACT. After excluding the patients with metastatic cancer at diagnosis, the patients treated with chemotherapy regimens other than the anthracycline/taxane-based, the patients treated with neoadjuvant radiation therapy, and the patients without available baseline blood counts, 120 patients were available for further study (**Figure 1**). **Table 1** shows clinical characteristics of those patients and histological features of their tumors. The median follow-up was 46 months [IC 95% (37–55)]. The median age at diagnosis was 56 years (range: 28 to 86). Almost all of the patients presented with infiltrating ductal carcinoma. The majority of the tumors were of intermediate or high histologic grade (40 and 55%, respectively). The prevalent tumor size and lymph node status at diagnosis were T2 (58%) and N0 (58%), respectively.

**Table 1:** Patient baseline characteristics (n = 120).

Variable	n (%)
**Age (years)**	
>50	79 (65.8%)
≤50	41 (34.2%)
**Histological type**	
Infiltrating ductal carcinoma	98 (81.7%)
Infiltrating and *in situ* ductal carcinoma	21 (17.5%)
Infiltrating and *in situ* lobular carcinoma	1 (0.8%)
**Histological grade (SBR grade)**	
1	4 (3.3%)
2	48 (40%)
3	66 (55%)
Unknown	2 (1.7%)
**HR expression**	
0%	110 (91.7%)
1–9% (low positive)	10 (8.3%)
**Ki67**	53.8 ± 28.8 (mean ± SD)
<20%	17 (14.5%)
≥20%	100 (85.5%)
**TNM classification**	
T1	30 (25%)
T2	70 (58.3%)
T3	13 (10.8%)
T4	5 (4.2%)
Tx	2 (1.7%)
**Inflammatory breast cancer**	5 (4.2%)
**Lymph node status**	
N0	69 (57.5%)
N1	35 (29.2%)
N2	4 (3.3%)
N3	3 (2.5%)
Nx	9 (7.5%)
**pCR**	34 (28.3%)
**TNM classification of surgical specimen**	
ypT1a ypN0	16 (13.3%)
ypT1b ypN0	8 (6.7%)
ypT0/T*is* ypN >0	8 (6.7%)
ypT1a ypN >0	3 (2.5%)
ypT1b ypN >0	3 (2.5%)
ypT1c ypN0	17 (14.2%)
ypT1c ypN >0	6 (5%)
ypT≥2 ypN0	7 (5.8%)
ypT≥2 ypN >0	18 (15%)

After NACT, 34 patients (28%) achieved pCR, whereas 24 patients presented with a small residual tumor (16 patients with ypT1a ypN0 and eight patients with ypT1b ypN0). Thus, 58 patients belonged to Group 1 and 62 patients to Group 2.

Out of 120 patients, 24 experienced distant recurrence.

### Higher TIL Counts Are Associated With Higher Chance of pCR

**Figure 2:**
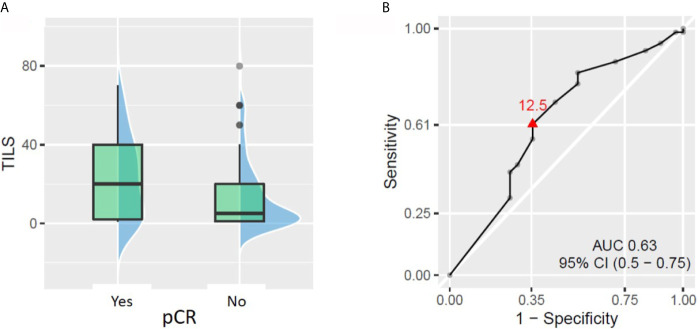
Relationship between tumor-infiltrating lymphocytes (TILs) on biopsy and pathologic complete response (pCR). **(A)** Patients who achieved pCR had significantly higher baseline TILs (p = 0.043). **(B)** ROC curve showing the best TIL cut-off to predict patient chances to reach pCR (TIL considered as a continuous variable).

TILs were assessed in pre-NACT tumor biopsy in 98 patients. The average and median values of TILs were 17.1 and 10% respectively (range: 1–80%). We observed a slightly significant correlation between baseline TIL levels as a continuous variable and pCR rate. The patients with TILs ≥12.5% had a 2-fold higher chance of achieving pCR than patients with TILs <12.5% (pCR rate 43.5% *vs* 21.0%, respectively, *p* = 0.029, **Figure 2**).When TILs were considered as a binary variable (high *vs* low, according to different prespecified cut-offs), only the cut-off at 30% showed a statistically significant difference in pCR rate between patient groups. The patients with ≥30% TILs had significantly higher pCR rate than the patients with <30% TILs (53.9% *vs* 23.6%, respectively, *p* = 0.007). However, the fraction [pCR + small residual tumors] was not significantly different between patients with baseline TILs ≥30 and <30%. No other TIL cut-off (5, 10 or 50%) was able to demonstrate a significant difference in pCR or [pCR + small residual tumor] rate.

### Low Baseline TIL Values Carry a Risk of Distant Recurrence

**Figure 3:**
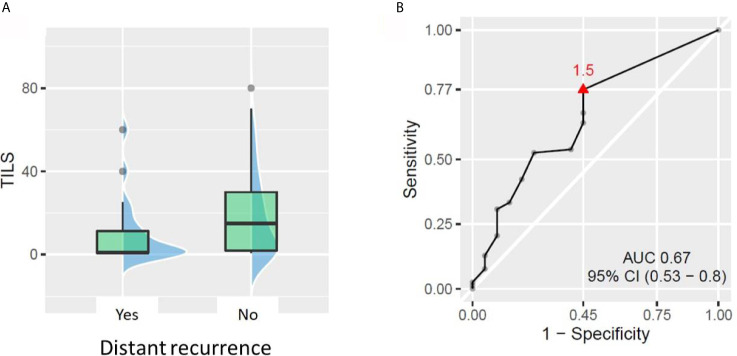
Relationship between tumor-infiltrating lymphocytes (TILs) and distant recurrence. **(A)** Patients with distant recurrences had lower baseline TILs (p = 0.021). **(B)** ROC curve showing the best TIL cut-off for predicting the patient risk of distant recurrence.

Out of the 98 patients with available TIL data, 20 patients experienced distant recurrence. The ROC curve approach showed that TIL value of 1.5% could statistically significantly discriminate between patients who developed distant recurrence and those who did not. The patients with less than 1.5% TILs in pre-NACT biopsy had almost 3-fold higher risk of developing distant recurrence than patients with ≥1.5% TILs (rates 37.9% *vs* 13%, respectively, *p* = 0.012, **Figure 3**). No other tested cut-off (5, 10, 30, and 50% TILs) rendered patient groups significantly different in terms of distant recurrence rate. Similarly, no significant difference in DRFS was demonstrated between patient groups, no matter which cut-off was used.

### Platelet-to-Lymphocyte Ratio Is the Only Blood Cell Count-Derived Parameter Associated With Response to NACT

**Figure 4:**
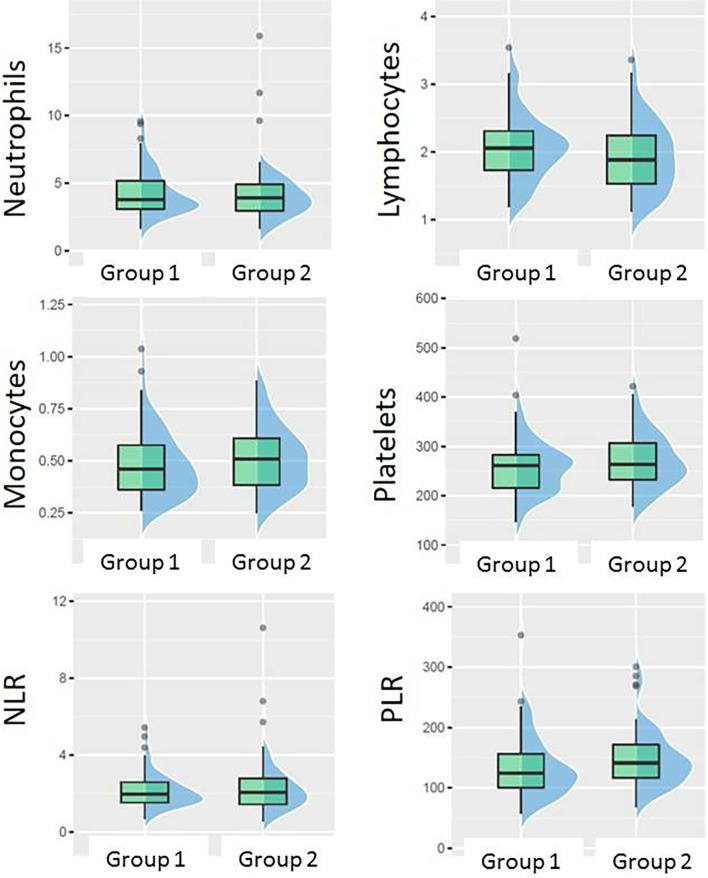
Boxplots showing the difference between Group 1 (pCR + small residual disease) and Group 2 (larger residual disease) in different blood counts or their ratios at baseline.

**Figure 5:**
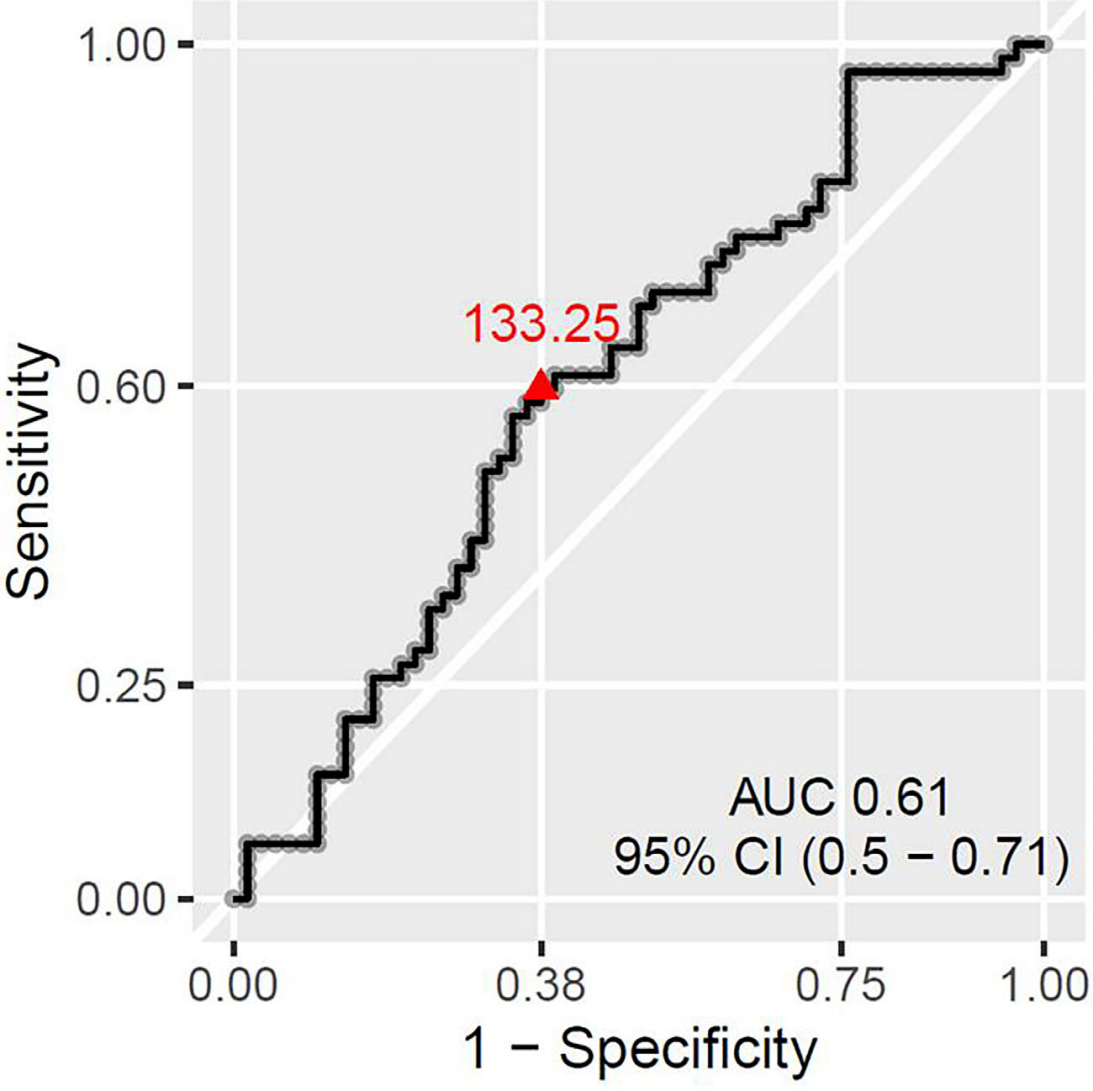
ROC curve showing the best PLR cut-off for predicting patient chances to have absent or small residual disease.

There was no statistically significant association between pCR and any of the absolute baseline blood cell counts (neutrophil, lymphocyte, monocyte, platelet count), or blood cell count-derived parameters (NLR, PLR). However, when favorable response was defined as [pCR + small residual disease] the only parameter significantly associated with that type of response was PLR. PLR was significantly lower in Group 1 (median 124.3, inter quartile range (IQR) 56) than in Group 2 (median 141.2, IQR 55), *p* = 0.045 (**Figure 4**). The ROC curve approach showed that the PLR cut-off at 133.25 best discriminates between favorable and unfavorable response patients (**Figure 5**).

### Relationship Between Blood Counts and Distant Recurrence Rate

No association could be established between baseline absolute neutrophil, lymphocytes, monocyte or platelet counts, as well as NLR or PLR, and distant recurrence rate or DRFS in our patient cohort.

### Relationship Between Blood Counts and TILs

In our cohort, no association could be found between baseline TIL levels and the baseline blood counts or blood count-derived parameters (NLR and PLR).

### Patient Group With Absent or Small Post-NACT Residual Disease Has Lower Risk of Distant Recurrence

No significant difference in the risk of distant recurrence could be found between pCR and non-pCR patients. However, in our cohort, the ensemble of pCRs and small residual post-NACT tumors (Group 1) was associated with lower risk of distant recurrence (p = 0.042). The relative risk of developing a distant recurrence was 2.3 times higher for patients in Group 2 (17 patients with metastases out of 62) than in Group 1 (seven patients with metastases out of 58).

### Patient Group With Absent or Small Post-NACT Residual Disease Have Longer DRFS

**Figure 6:**
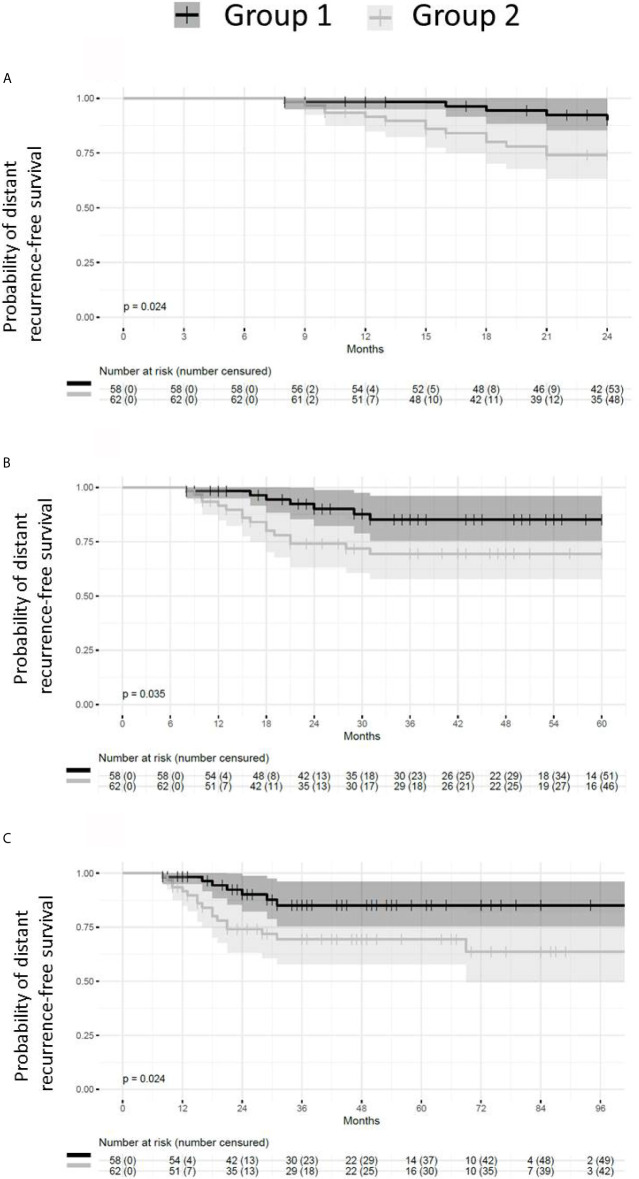
Probability of distant recurrence-free survival from diagnosis. Patients with pCR or small residual disease (Group 1) after neoadjuvant chemotherapy exhibit better distant recurrence-free survival (DRFS) than patients with large residual disease (Group 2) (p = 0.024). **(A)** DRFS from diagnosis to 24 months (p = 0.024). **(B)** DRFS from diagnosis to 60 months (p = 0.035). **(C)** DRFS from diagnosis to 96 months (p = 0.024).

**Figure 7:**
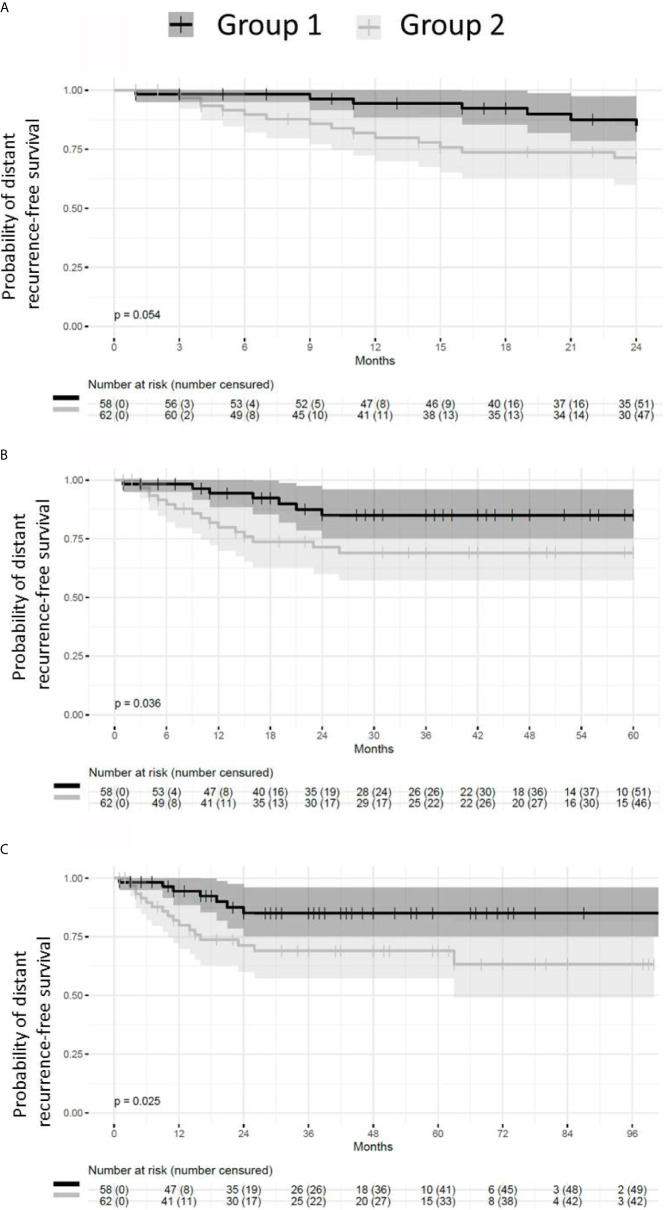
Probability of distant recurrence-free survival from the time of surgery. Patients with pCR or small residual disease (Group 1) after neoadjuvant chemotherapy exhibit better distant recurrence-free survival (DRFS) than patients with large residual disease (Group 2). **(A)** DRFS from the time of surgery to 24 months (p = 0.054). **(B)** DRFS from the time of surgery to 60 months (p = 0.036). **(C)** DRFS from the time of surgery to 96 months (p = 0.025).

Patients presenting with small or absent residual disease after NACT also had longer DRFS than the patients with larger residual disease (**Figure 6**). The difference between the two groups remained statistically significant for DRFS at 24, 60 and 96 months after diagnosis (*p* = 0.024, 0.035 and 0.024 respectively). A statistically significant difference in DRFS was also observed at 60 and 96 months, but not at 24 months after surgery (*p* = 0.036, 0.025 and 0.054 respectively) (**Figure 7**).

Taken together, these results show that baseline counts of white blood cells (neutrophils, lymphocytes, monocytes) or platelets, as well as the derived parameters (NLR, PLR) do not have higher pCR predictive value than TILs. Similarly, the evaluated blood parameters were not stronger biomarkers of distant recurrence than TILs. In this TNBC cohort, pCR status was not associated with the risk of recurrence or DRFS. However the group comprised of the patients with pCR and the patients with small residual tumor had lower risk of distant recurrences and better DRFS than the patients with larger residual disease. PLR was the only parameter which significantly differed between these two groups of patients.

## Discussion

In this retrospective study, we first showed that baseline TILs were better biomarkers of TNBC response to standard NACT than any of the blood parameters studied (neither pre-treatment counts of neutrophils, lymphocytes, monocytes or platelets nor the derived parameters, NLR and PLR). This was observed when pCR was used as the criterion of favorable response to NACT. However, when both pCR (ypT0/is ypN0) and the presence of small residual tumor (ypT1a/b ypN0) were considered as favorable response, the only predictive biomarker was a blood cell count-derived one, the PLR.

This loss of predictive value for TILs but gain for PLR has not been reported before. One could argue that the more convincing findings are the ones obtained when pCR was used as the criterion of good response. pCR is nowadays a universally accepted strong predictor of good outcome of TNBC patients (34), however it has been evoked that persistence of a minimal residual disease in TNBC offers the patients practically identical outcome as the pCR status. Namely, Symmans et al. proposed a histology-based measure of post-NACT breast disease, the Residual Cancer Burden (RCB) and showed that patients with RCB-0 (pCR) and RCB-I (minimal residual disease) have the same 5-year prognosis (9). We could not assess RCB in all our patients due to a lack of tissue material for some of them. For that reason, we arbitrarily defined the status ypT1ab ypN0 as small residual post-NACT disease. In our cohort, the fraction of patients with small residual disease was quite large (20% of the entire cohort and 28% of the non-pCR patients), out of which only 2 (8.3%) experienced distant recurrence (data not shown). The recurrence rate of the patients left with a small residual tumor was almost 2-fold lower than the recurrence rate of the patients with pCR (5/35, 14.7%, data not shown). Moreover, these characteristics of the pCR patients and the patients with small residual tumors were the same in the subpopulation for which we had baseline TIL data (98 out of 120 pts): 31 pCR patients, out of which three recurred (9.6%), and 19 patients with small residual tumors out of which only one recurred (5.2%) (data not shown). This demonstrates that our cohort actually had many very good responders in the non-pCR group, the patients with the ypT1a/b ypN0 status. The first possible explanations why TILs lost their predictive power when the good response group was enlarged by the patients with small residual tumors after NACT could be a particular intrinsic sensitivity to NACT of the patients with small residual disease and a development of TILs during NACT. In other words, these patients might have had lower baseline TILs, but increase their number during NACT and present only a small residue (near-pCR status) at the end of the treatment. Indeed, the patients left with small residual disease post-NACT, in our cohort, had significantly lower baseline TILs (median: 2, mean ± SD: 14 ± 7) than the patients with pCR status post-NACT (median: 20, mean ± SD: 23 ± 21) (p <0.05, data not shown). The activation of the anti-tumor immune response during NACT is the basis of the cancer-immunity cycle and is actually initiated by destruction of the tumor cells sensitive to chemotherapy. This destruction liberates tumor neoantigens and damage-associated molecular pattern (DAMP) signals, thus attracting lymphocytes to the tumor bed. This phenomenon is the major factor responsible for good cancer response to cytotoxic chemotherapy and good long-term prognosis of cancer patients (35). Many studies, starting with the seminal work of Demaria et al. (36), have shown that TILs can develop during NACT (37). In some of them, like the one of Jovanovic et al., the pre-treatment TILs were not predictive at all, however mutations in the DNA damage response-related genes were, which indicates that TNBCs, as well as other cancers, may have an intrinsic sensitivity to DNA-damaging agents, which is behind a good response to DNA-damaging agents and the consequent TIL development (38).

The strong predictive value of TILs in TNBC has been reported in numerous studies, however, in most of them the cut-offs of 50 or 60% best separated good responders (pCR) from the poor ones (non-pCR) (39, 40). In our study, 30% was the best-separating among the predefined cut-offs (5, 10, 30, and 50%), similarly to what was reported by Ono et al. (41). Some of the recent studies showed that lower cut-offs than the ones used for definition of lymphocyte-predominant breast cancer (≥50%) can also statistically significantly separate patient groups according to the pCR rate (42–45).

The presence of TILs in tumors reflects both systemic and local immunity (within the tumor bed). It has not been fully explored yet how the parameters of systemic immunity (white blood cell counts, circulating immune cell subpopulations) correlate with lymphocytic infiltration of cancers, including breast cancer. We did not find a correlation between any baseline blood count or blood count-derived parameter with TILs, which might indicate a distinct regulation of systemic and tumor-site immune response. Previous studies in metastatic breast cancer have shown inferior patient outcomes in patients with general or specific (CD4+) lymphopenia, however data on baseline blood count impact on early TNBC response to therapy and outcome are still scarce (46, 47).

To the best of our knowledge, this is the largest study of NACT-treated TNBC patients which showed predictive value of PLR. Graziano et al. found that a combination of low NLR (≤2.42) and low PLR (≤104.47) was associated with higher pCR rate in a population of 373 all-type breast cancer patients, including only 62 TNBC patients (6). Cuello-Lopez et al. showed that low pre-treatment PLR (PLR <150) correlated with higher pCR rate in 272 breast cancer patients, however, no correlation could be found within the 62 TNBC patients of their cohort (25). Asano et al. observed similar results in a population of 177 breast cancer patients, but only 61 of them had a TNBC (23). In this study, low PLR was defined as lower than 133.25, which is similar to the published data (25, 48). The particularity of our study is that low PLR was not significantly associated with pCR only but with pCR and the presence of small post-NACT residual tumor. As we already explained above, the group of patients with small residual tumours after NACT had an even better outcome than the pCR group, so PLR was the only predictor of the real good response in our cohort. Indeed, and contrary to TILs, PLR was practically identical in the pCR group (median: 125.3, mean ± SD: 131.5 ± 46.3) and the small residual disease group (median: 124.3, mean ± SD: 139.4 ± 62.4) (data not shown). One explanation for this superiority of PLR over TILs could be that PLR did not change the same way as TILs during NACT. TILs could have developed/increased during NACT, and thus contribute to good response even in the patients with low pre-treatment TIL values. PLR could have had dynamics with less change impacting the response. Studies about PLR dynamics during NACT are scarce; most of them were performed in esophageal or rectal cancer treated by chemoradiation and their results are conflicting. Some studies reported the association of PLR increase and pCR, whereas in others lowering of PLR was favorable for good response (49, 50). To better determine the time point during NACT at which the PLR value is most predictive, as well as to determine the predictive value of PLR change between different time-points during NACT, prospective studies are needed, like the one our group has recently initiated (PERCEPTION trial, NCT04068623) (51).

We also showed prognostic value of TILs, with regards to patient DRFS. The cut-off revealed by the ROC curve approach as the best separator between the patients at risk of distant recurrence and the ones without was quite low (1.5%) and ‘artificial’ in a certain sense (the value of 1.5% TILs cannot be obtained at TIL assessment). However, it could be viewed as an equivalent of the cut-off zero (presence/absence of TILs), indicating that TNBC totally devoid of TILs (the so-called immune-deserted type) have the worst outcome. One strong reason why the cut-off for TILs as prognosticators was so low in our study is the specificity of the analyzed cohort (selection bias). The fraction of patients with metastatic recurrence was relatively low (20%) and the DRFS/OS relatively long. It may suggest that our cohort was composed of patients with less aggressive TNBCs; for example, almost half of the patients (45%) had tumors of intermediate/low grade, which also could have more indolent clinical course.

Although PLR was well associated with good response to NACT, it was not a prognostic biomarker in our study. As we showed that the ensemble of patients with pCR and small residual disease had better outcome than the rest of the patients, and that PLR was predictive of that type of response, we are hypothesizing that a prognostic value of PLR could be confirmed in a larger study. Corbeau et al. demonstrated that higher PLR is associated to worse RFS and OS in 280 breast cancer patients, 72 of which were TNBC (52). One recent meta-analysis found that higher PLR was associated to worse disease-free and overall survival in all-type breast cancer. Given that a few TNBC studies were included in this meta-analysis and their small size, no significant results could be established in TNBC (53). Other researchers have shown prognostic value of low PLR (<100–190). For example, Huszno et al. observed that low PLR was associated to improved OS in 86 TNBC patients (48). Studies have also been conducted on metastatic TNBC. Vernieri et al. found that PLR ≥200 was associated with worse progression-free survival, but not with overall survival in 57 metastatic TNBC patients (54).

Besides their essential roles in hemostasis, platelets also support cancer progression and metastatic development, through the production and excretion of platelet-derived growth factors (PDGF A and B), platelet-derived angiogenesis factors and vascular endothelial growth factor A (VEGF-A), as well as by interacting with interleukins and myeloid metalloproteins (25, 55). PDGF is also produced and excreted by cancer cells, stimulating tumor progression and dissemination in an autocrine manner (16, 56). On the other hand, the release of inflammatory mediators, such IL-1, IL-3 and IL-6 by cancer cells, triggers the differentiation of megakaryocytes into platelets (57), thus fueling a vicious cycle where platelets and tumor cells continuously stimulate each-other’s growth. Romero-Cordoba et al. showed that PDGF are involved in the regulation of transmembrane proteins, such as CUG domain-containing protein 1 (CDCP1), which is overexpressed in TNBC and stimulates tumor progression (57). An increase in platelets can also lead to disseminated intravascular coagulation, favoring tumor metastasis in different cancers, such as gastric, colon, lung, kidney and prostate (58). We may therefore hypothesize that high PLR reflects a systemic status with more tumor-promoting and less tumor-suppressing action (by platelets and lymphocytes, respectively), allowing metastatic progression of TNBC.

A great majority of the studies assessing circulating blood cells and platelets as predictive or/and prognostic biomarkers in TNBC demonstrated stronger prognostic and predictive value of NLR (26, 27, 48, 59–63). These studies have been carried out either in the adjuvant setting or in less than 90 TNBC patients treated by NACT. In our cohort of 120 TNBC patients, no correlation was observed between the baseline NLR and response to NACT. Likely it is again the selection bias which is behind this finding. It should be noted that 95% of the analysed patients had normal neutrophil, lymphocytes and platelet counts before NACT, so we did not have many extremely high or low values for NLR or PLR, which likely was one of the major reasons for the lack in statistically significant differences between patient groups.

Our study has several limitations. It is a monocentric non-consecutive retrospective study with a relatively small cohort size. The peripheral blood cell counts can be affected by nutrition, medication and underlying medical conditions, such as infection or other diseases. Therefore, the findings of our study need to be validated in larger patient cohorts. In this line, our group has recently started a prospective translational trial, PERCEPTION, whose main objective is evaluation of predictive and prognostic value of blood cell counts, NLR, PLR and TILs, before, during and after anthracycline/taxane-based NACT in TNBC patients (51).

## Conclusion

This study confirms the predictive and prognostic value of TILs in TNBC patients treated by standard neoadjuvant chemotherapy; however the study also shows that they are not a perfect predictor of good response. In addition, in real-life situations, TILs might be difficult to assess if a patient is coming to a tertiary cancer care center after being diagnosed outside. In such situations, PLR might be a good orientation about the patient chance to respond well to NACT, either reaching pCR or the status with a small residual disease.

Our findings need to be validated both in larger retrospective studies, on cohorts similar to ours in terms of patient response, as well as in prospective studies. Compared to TILs, a peripheral blood examination is simpler, less expensive and less invasive and thus very suitable for dynamic assessment. Therefore, identifying reliable biomarkers from peripheral blood cell count should be continued, optimized and standardized, in order to introduce these easy-to-assess parameters into clinical practice. As TILs remain to be useful biomarkers in TNBC management, efforts should be intensified to introduce them into the routine practice, including the organizational adjustment which facilitates access to the pre-treatment tumor tissue samples.

## Data Availability Statement

The raw data supporting the conclusions of this article will be made available by the authors, without undue reservation.

## Ethics Statement

Ethics approval of the study was obtained from the Ethical Committee of the Clinical Investigation Centre of the Rhone-Alpes-Auvergne region, Grenoble, France; approval number: IRB 5921. Written informed consent for participation was not required for this study in accordance with the national legislation and the institutional requirements.

## Author Contributions

NR-R and CA conceived the study and contributed equally to this work. SL and NR-R performed the literature research. NR-R, CA, XD, M-AM-R, FP-L, and SL designed the study. SL collected all the data and wrote the first draft of the manuscript. MK and NL performed the TILs assessment and contributed equally to this work. IM performed the statistical analysis. CA, NR-R, XD, IM, M-AM-R, and FP-L revised the article. All authors contributed to the article and approved the submitted version.

## Conflict of Interest

The authors declare that the research was conducted in the absence of any commercial or financial relationships that could be construed as a potential conflict of interest.
